# Allergy to cow's milk protein and reaction to methylprednisolone – case study

**DOI:** 10.1186/1939-4551-8-S1-A206

**Published:** 2015-04-08

**Authors:** Isabela Mina, Fernanda Aleixo Teixeira, Kaila Barroso De Andrade Medeiros, Brunna De Freitas Castello, Phelipe Souza, Patricia Stefanelli, Persio Roxo Jr, Clara Lima Santis

**Affiliations:** 1 Clinical Hospital of Ribeirão Preto Medical School, Brazil; 2 School of Medicine of Ribeirao Preto - University of Sao Paulo, Brazil

## Background

Certain foods are used as excipients in pharmacological products. Thus, patients with lgE-mediated food allergy could present reaction to the pharmacological product whose the excipient is the food in question. Such reactions are rare, considering the small quantity of food protein present in the products. In most situations, these medicines need not be avoided in patients with food allergy, as most tolerate the medication without complications. Reactions to the drugs in patients with allergy to cow’s milk protein (CMPA) are scarcely reported. Generally the lactose is not contaminated with milk protein, but this could happen in rare situations. There are reports of two patients with high levels of lgE to cow’s milk that presented urticarial after receiving methylprednisolone.

## Methods

A case report of a seven-year-old boy with CMPA that presented reaction when received endovenous methylprednisolone.

## Results

A male patient, seven years old, previously diagnosed with asthma, rhinitis allergy and encephalopathy after anaphylactic shock due to dipyrone, received a diagnosis of CMPA at 5 months old, when presented urticaria and vomiting immediately after the use of child formula based on cow’s milk, having undergone total exclusion of the food and its derivatives thereof. Laboratory Tests: lgE to casein: 6,45 KU/L; lgE to beta-lactoglobulin: 5,25 KU/L; lgE to alpha-lactalbumin: 77 KU/L; lgE to cow’s milk: > 100 KU/L. Evolution: At age 7, during hospitalization for bronchospasm and pneumonia, the patient received endovenous methylprednisolone and presented diffuse urticarial lesions, angioedema and respiratory difficulty, starting immediately after the infusion.

## Conclusions

Test performed with injectable methylprednisolone revealed the presence of nanograms of beta-lactoglobulin in the medication. The presence of food protein, intentionally or by contamination, in medications whose excipients are derived from food, makes the patients with food allergy, susceptible to present adverse reaction to the medication in question, although this occurrence is rare. Therefore patients with CMPA should be advised regarding the use of medications containing milk protein.

